# Fulvestrant-Induced Cell Death and Proteasomal Degradation of Estrogen Receptor α Protein in MCF-7 Cells Require the CSK c-Src Tyrosine Kinase

**DOI:** 10.1371/journal.pone.0060889

**Published:** 2013-04-04

**Authors:** Wei-Lan Yeh, Keiko Shioda, Kathryn R. Coser, Danielle Rivizzigno, Kristen R. McSweeney, Toshi Shioda

**Affiliations:** Center for Cancer Research, Massachusetts General Hospital Cancer Center and Harvard Medical School, Charlestown, Massachusetts, United States of America; II Università di Napoli, Italy

## Abstract

Fulvestrant is a representative pure antiestrogen and a Selective Estrogen Receptor Down-regulator (SERD). In contrast to the Selective Estrogen Receptor Modulators (SERMs) such as 4-hydroxytamoxifen that bind to estrogen receptor α (ERα) as antagonists or partial agonists, fulvestrant causes proteasomal degradation of ERα protein, shutting down the estrogen signaling to induce proliferation arrest and apoptosis of estrogen-dependent breast cancer cells. We performed genome-wide RNAi knockdown screenings for protein kinases required for fulvestrant-induced apoptosis of the MCF-7 estrogen-dependent human breast caner cells and identified the c-Src tyrosine kinase (CSK), a negative regulator of the oncoprotein c-Src and related protein tyrosine kinases, as one of the necessary molecules. Whereas RNAi knockdown of CSK in MCF-7 cells by shRNA-expressing lentiviruses strongly suppressed fulvestrant-induced cell death, CSK knockdown did not affect cytocidal actions of 4-hydroxytamoxifen or paclitaxel, a chemotherapeutic agent. In the absence of CSK, fulvestrant-induced proteasomal degradation of ERα protein was suppressed in both MCF-7 and T47D estrogen-dependent breast cancer cells whereas the TP53-mutated T47D cells were resistant to the cytocidal action of fulvestrant in the presence or absence of CSK. MCF-7 cell sensitivities to fulvestrant-induced cell death or ERα protein degradation was not affected by small-molecular-weight inhibitors of the tyrosine kinase activity of c-Src, suggesting possible involvement of other signaling molecules in CSK-dependent MCF-7 cell death induced by fulvestrant. Our observations suggest the importance of CSK in the determination of cellular sensitivity to the cytocidal action of fulvestrant.

## Introduction

Approximately 70% of breast cancers express estrogen receptor α (ERα), and most of these ERα-positive primary tumors depend on estrogen signaling for their growth and survival [1]. Endocrine therapy aims to shut off estrogen signaling in ERα-positive breast cancer cells to halt cell proliferation and/or to induce cell death [2]–[7]. Two types of antiestrogens with distinct mechanisms of actions have been used for this purpose: Selective Estrogen Receptor Modulators (SERMs) and the Selective Estrogen Receptor Down-regulators (SERDs). The SERMs, represented by tamoxifen or raloxifene, bind to ERα as partial agonist or antagonists in a manner dependent on target tissues [8]–[10]. On the other hand, the SERDs, represented by fulvestrant, bind to ERα and induce rapid proteasomal degradation of ERα protein [11]. Unfortunately, the benefit of endocrine therapy is seriously limited by resistance of tumors against antiestrogens [12], and a large number of studies have proposed molecular mechanisms behind the endocrine therapy resistance of human breast cancer cells. When activated by agonistic ligands, ERα functions as a transcription factor and affects expression of thousands of genes in human breast cancer cells [13]–[15]. In addition, ERα initiates rapid intracellular signaling [16] through phosphorylation of membrane receptor kinases, including insulin-like growth factor I receptor (IGF-IR) [17], epidermal growth factor receptor (EGFR) [18], and HER2/ERBB2 [19]. ERα also interacts with other signaling kinases and adaptor molecules such as c-Src [20], Shc [21], PAK1 [22], DLC1 [23], [24], PELP1/MNAR [22], [25], [26], and p85 PI3-kinase regulatory subunit [27]. These interactions lead to activation of downstream signaling kinases such as the p42/44 MAPK and AKT [28], which play critical roles in regulating cell proliferation and survival. Some of these ERα-activated protein kinases (e.g., c-Src, PAK1, MAPK, and AKT) phosphorylate ERα to enhance the genomic actions of ERα. Roles of another network of signaling pathway involving STAT1, interferon regulatory factor 1, NF-κB, and their downstream effectors (e.g., caspases and BCL2 family apoptosis regulators) are also becoming increasingly evident [29]. Thus, a large body of evidence supports the notion that a highly complex signaling network is involved in the mechanism of estrogen actions and possibly the endocrine therapy resistance of ERα-positive breast cancer cells.

To identify novel components in the signaling network leading to endocrine therapy resistance, functional screening studies using the RNAi knockdown technique have been performed by several laboratories. For example, Iorns et al. [30] transfected MCF-7 human breast cancer cells with an arrayed library of siRNA oligonucleotides that targeted 779 human kinases and phosphatases. By exposing cells to tamoxifen and identifying drug-resistant clones, they identified three protein kinases (CDK10, CRK7, and MAP2K7) required for tamoxifen-induced cell death. Taking a similar approach of Iorns et al., in the present study we performed lentivirus-based RNAi knockdown screening experiments covering the entire human kinases and phosphatases and identified CSK (c-Src tyrosine kinase) as a novel signaling molecule required for fulvestrant-induced MCF-7 cell death. Whereas RNAi knockdown of CSK caused significant resistance to fulvestrant, it did not affect sensitivities to either tamoxifen or paclitaxel. We provide evidence that this strong specificity of fulvestrant resistance caused by CSK knockdown was due to suppression of the fulvestrant-induced proteasomal degradation of ERα protein, which is not involved in the mechanisms of actions of tamoxifen or paclitaxel. Our present study provides important insights into the molecular mechanisms of the cytocidal action of fulvestrant in human breast cancer cells, providing evidence of requirement of CSK.

## Results

### RNAi knockdown of the c-Src Tyrosine Kinase (CSK) caused resistance of MCF-7 cells to fulvestrant

**Figure 1:**
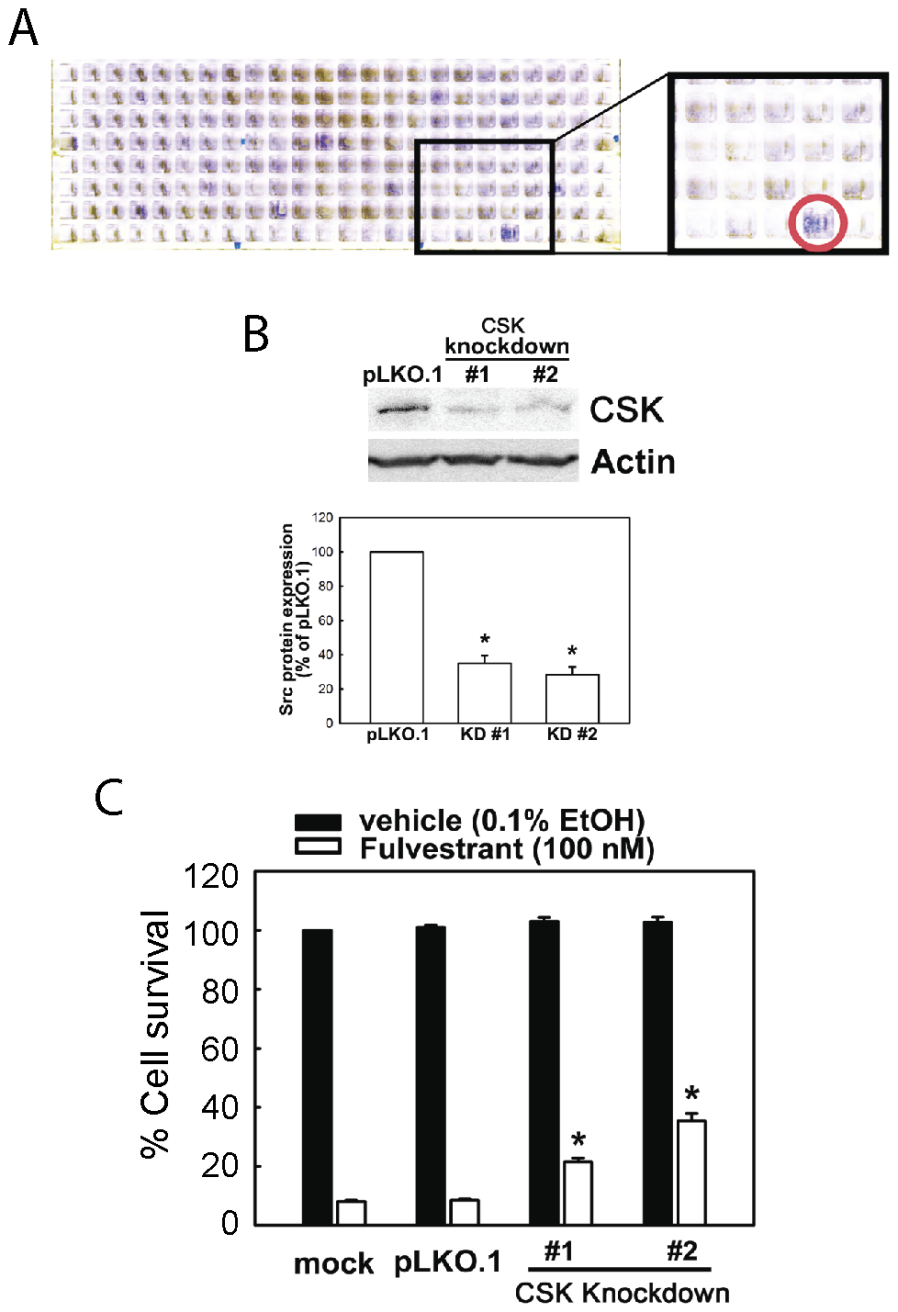
RNAi knockdown of CSK in MCF-7 cells causes resistance to fulvestrant. (A) RNAi knockdown screening reveals dependence of fulvestrant-induced MCF-7 cell death on CSK. Cells infected with lentiviruses expressing shRNA to CSK (well is identified by red circle) survived after 7 days of exposure to 100 nM fulvestrant. Crystal violet staining of a representative screening plate is shown. (B) Knockdown of CSK protein expression by shRNA lentiviruses. Cells were infected with empty lentivirus vector (pLKO.1) or two independent clones of lentiviruses expressing different shRNA species targeting CSK shown in Figure 1 (CSK KD#1 and #2) and subjected to Western blotting quantitation of CSK protein expression (top). Intensities of CSK protein bands were determined by densitometry as shown in the bar graph (bottom, mean±SEM of three independent experiments. Asterisk indicates statistical significance, p<0.05). (C) Infection by lentiviruses expressing shRNA targeting CSK causes fulvestrant resistance of MCF-7 cells. Cells were infected with empty lentivirus vector (pLKO.1) or two independent clones of lentiviruses expressing different shRNA species targeting CSK (CSK KD #1 and #2) and exposed to fulvestrant, or vehicle for 7 days. % Cell survival (mean±SEM) was determined by three independent experiments. *, p<0.001 to both mock infected and pLKO.1-infected controls exposed to fulvestrant). No significant changes were observed with cell survival ratio of any virus-infected cells compared to mock infected control.

Our prior studies revealed the critical importance of BIK (a BH3-only family pro-apoptotic protein) and TP53 (a tumor suppressor transcription factor necessary for transcriptional induction of the BIK mRNA transcripts) in fulvestrant-induced apoptosis of MCF-7 cells [31], [32]. To obtain further insights into the mechanism of fulvestrant actions, we performed RNAi knockdown screenings to identify additional molecules required for fulvestrant-induced MCF-7 cell apoptosis. MCF-7 cells grown in 384-well plates were infected with a library of arrayed lentiviruses expressing shRNA species targeting the entire RefSeq collection of know human protein kinases and phosphatases consisting of 6,560 lentivirus clones [33], [34]. Cells were then exposed to 100 nM fulvestrant for 7 days, and surviving cells were visualized by crystal violet staining. These screenings revealed that RNAi knockdown of MAP2K7 or CSK (c-Src tyrosine kinase, NCBI gene ID = 1445) strongly suppress fulvestrant-induced MCF-7 cell death (Fig. 1A for CSK data; MAP2K7 data not shown). Since a similar RNAi knockdown project by Iorns et al. already identified MAP2K7 and several other kinases including CDK10 as Ser/Thr kinases required for tamoxifen sensitivity of MCF-7 cells, we focused on the roles of CSK in the cytocidal action of fulvestrant on MCF-7 cells.

RNAi knockdown of two independent shRNA lentivirus clones targeting human CSK [The RNAi Consortium Clone ID = TRCN0000199018 (target sequence, 5′-CCACTAAGTCTGACGTGTGGA, is in the CSK coding sequence) and TRCN0000199031 (target sequence, 5′-CCGTCTCTCTTGGACCCACCT, is in the 3′-UTR of the CSK mRNA transcripts); hereafter referred to as shRNA #1 and #2, respectively] confirmed the requirement of CSK for the cytocidal action of fulvestrant in MCF-7 cells. When cells were infected with these shRNA lentiviruses at MOI = 4∼8 and selected by puromycin resistance for 48 hours, we observed about 65%–75% reduction in CSK protein expression (Fig. 1B). The CSK RNAi knockdown was stable in the infected cells for at least five passages, within which all experiments in the present study were performed. Exposure of cells to 100 nM fulvestrant for 7 days induced massive cell death in mock-infected cells and cells infected with the pLKO.1 empty lentiviral vector resulted in only 8.1±0.3% and 8.5±0.6% surviving cells, respectively (Fig 1C and Fig. S1). In contrast, MCF-7 cells infected cells the CSK shRNA lentiviruses showed significant resistance to fulvestrant-induced death, with 21.5±1.3% and 35.3±2.7% surviving cells after exposure to shRNA #1 and #2, respectively.

To determine whether the CSK knockdown efficiency correlates with the strength of fulvestrant resistance, MCF-7 cells were infected with a 10-clone panel of shRNA lentiviruses (Table S1), and their fulvestrant-induced cell death was examined (Fig. S2). Effective RNAi knockdown of CSK was observed with four shRNA lentiviral clones whereas three clones as well as pLKO.1 control clones failed RNAi knockdown. Fulvestrant resistance was observed with the four shRNA lentiviral clones that effectively knocked down CSK whereas cells infected with the failed lentiviral clones or the pLKO.1 empty viral vector control were completely killed after 7-day exposure to 100 nM fulvestrant. These results indicate that CSK is required for fulvestrant-induced MCF-7 cell death.

### RNAi knockdown of CSK does not affect MCF-7 cell sensitivity to either tamoxifen or paclitaxel

**Figure 2:**
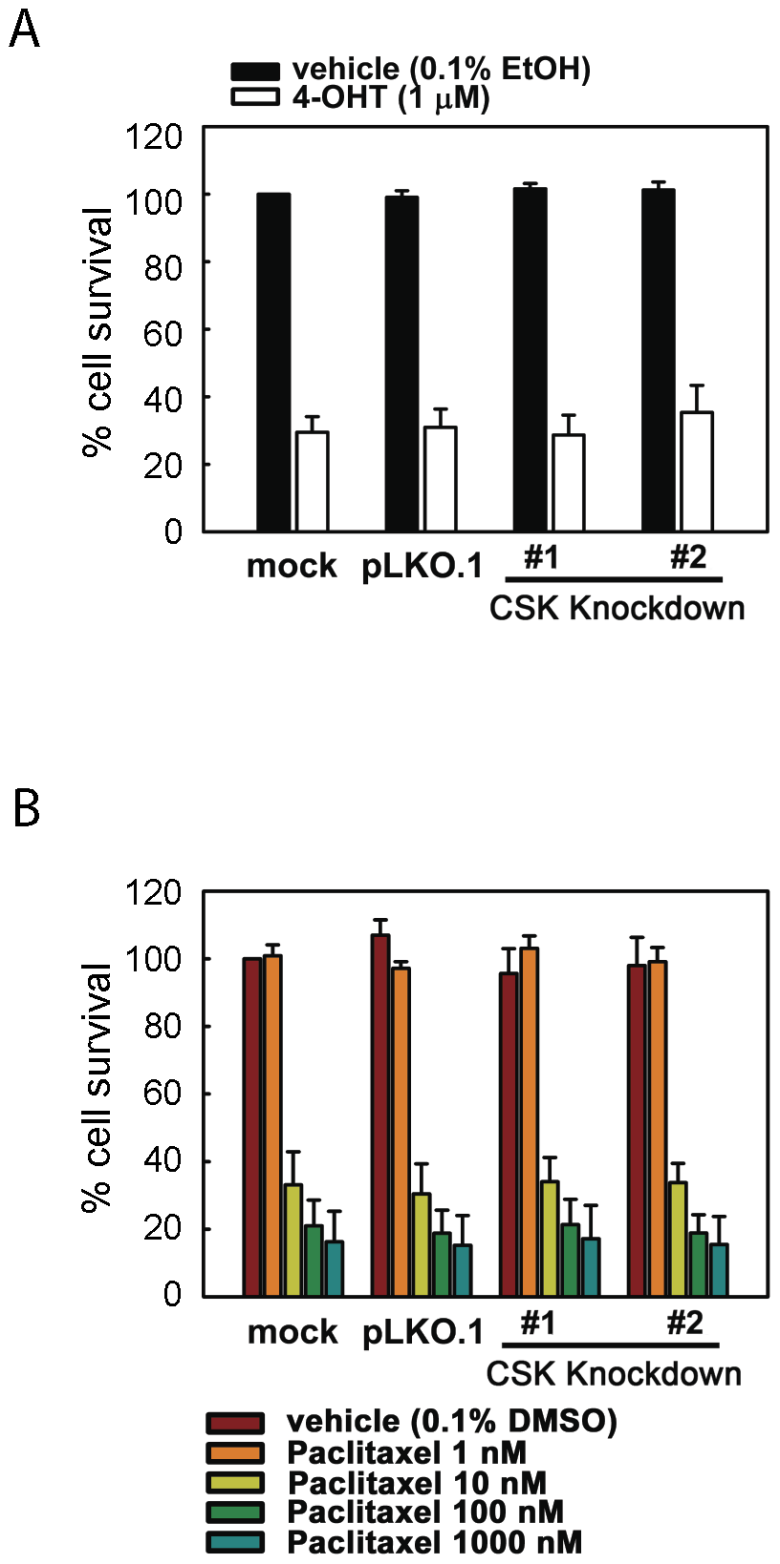
RNAi knockdown of CSK does not affect MCF-7 cell sensitivity to tamoxifen or paclitaxel. Cells were infected with empty lentivirus vector (pLKO.1) or two independent clones of lentiviruses expressing different shRNA species targeting CSK shown in Figure 1 (CSK KD#1 and #2) and then exposed to 1 µM 4-hydroxytamoxifen (4-OHT) for 10 days (A) or 1–1000 nM paclitaxel for 2 days (B). Cell viability was determined by crystal violet staining (Fig. S3) and quantified by spectrophotometry (mean±SEM of three or more independent experiments).

Two different types of antiestrogens are presently used for endocrine therapy of breast cancer–namely, the SERDs (represented by fulvestrant) and the SERMs (represented by tamoxifen). Cross-resistance of breast cancer cells to these distinct types of drugs is often observed, in both clinical and cell culture settings [35]–[37]. To examine whether CSK is required for the cytocidal effects of tamoxifen, MCF-7 cells were exposed to 4-hydroxytamoxifen (4-OHT), which is the biologically active metabolite of tamoxifen [38]. A 10-day exposure to 1 µM 4-OHT caused significant MCF-7 cell death although its cytocidal effect was weaker than that of fulvestrant (Figs. 2A and S3A), in agreement with previous studies [39], [40]. To our surprise, RNAi knockdown of CSK did not affect the tamoxifen effect at all. These results indicate that CSK is specifically required for fulvestrant (SERD)-induced MCF-7 cell death while it is dispensable for the cytocidal action of tamoxifen (SERM).

**Figure 3:**
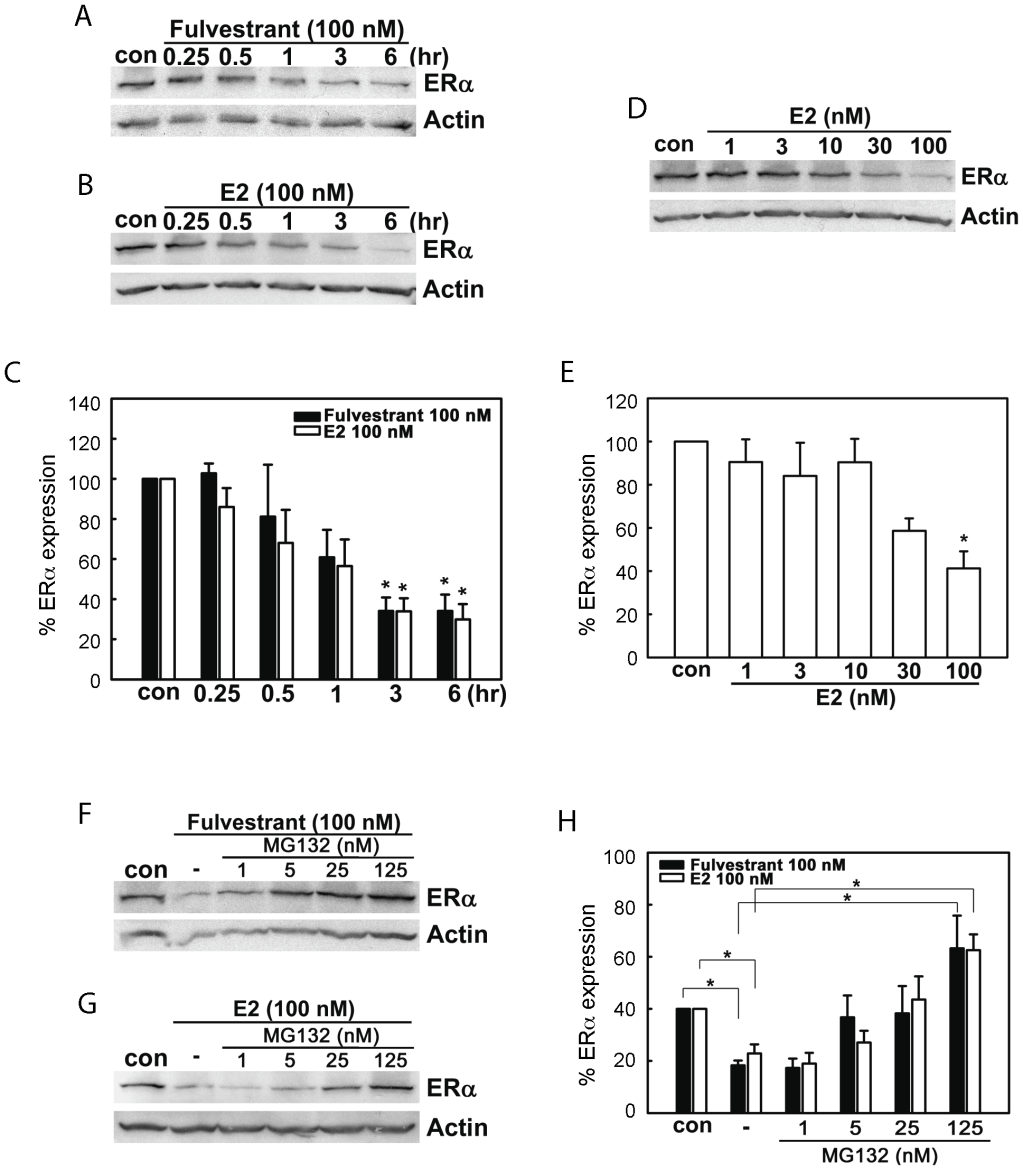
Both fulvestrant and 17β-estradiol (E2) enhance proteasomal degradation of ERα protein in MCF-7 cells. (A–C) Fulvestrant (A) and E2 (B) caused time-dependent reduction in ERα protein expression: Western blotting. Intensities of ERα protein bands were determined by densitometry (C, mean±SEM of three independent experiments. Asterisks indicate statistical significance, p<0.05 to vehicle control). (D, E) E2 dose-dependent reduction in ERα protein expression. Cells were exposed to varying concentrations of E2 for 6 hours and subjected to Western blotting analysis of ERα protein (D). Intensities of ERα protein bands were determined by densitometry (E, mean±SEM of three independent experiments. Asterisk indicates t-test significance p<0.05 to vehicle control). (F–H), Pre-exposure to MG132 dose-dependently prevented reduction in ERα protein expression caused by fulvestrant (F) and E2 (G). Con, vehicle control (0.1% ethanol). Cells were exposed to varying concentrations of MG132 for 30 minutes and then exposed additionally to fulvestrant or E2 for 6 hours. Intensities of ERα protein bands were determined by densitometry (H, mean±SEM of three independent experiments. Asterisks indicate statistical significance, p<0.05).

To further characterize the specificity of the CSK requirement for drug-induced MCF-7 cell death, we examined the effects of RNAi knockdown of CSK on MCF-7 cell sensitivity to paclitaxel, a widely used chemotherapeutic drug that inhibits dissociation of microtubule polymers [41]. A 2-day exposure of MCF-7 cells to varying concentrations of paclitaxel (1–1000 nM) caused massive cell death in a dose-dependent manner (Figs. 2B and 3SB). However, RNAi knockdown of CSK failed to affect the cytocidal effects of paclitaxel. Thus, the drug resistance of MCF-7 cells infected with shRNA lentiviruses targeting CSK was highly specific for fulvestrant.

### CSK is required for fulvestrant-induced ERα protein degradation in estrogen-dependent human breast cancer cells

Fulvestrant causes proteasomal degradation of ERα protein in breast cancer cells [11], [31], [35]. High concentrations of 17β-estradiol (E2), a physiological ligand of ER, also causes proteasomal degradation of liganded ERα protein [42]–[44]. Since strong genetic and phenotypic heterogeneity, including sensitivity to antiestrogens, has been shown to occur in MCF-7 cell cultures maintained in different institutions and cell resource repositories [45]–[50], we first attempted to confirm that both fulvestrant and E2 cause proteasome-dependent degradation of ERα protein. When MCF-7 cells were exposed to 100 nM fulvestrant, expression of ERα protein was reduced in a time-dependent manner (Fig. 3A, 3C). Similarly, exposure of hormone-starved MCF-7 cells to 100 nM E2 caused time-dependent reduction in ERα protein expression (Fig. 3B, 3C). Under our experimental conditions, the time-dependent reduction in ERα protein caused by exposure to fulvestrant and E2 were comparable, with only 35% of ERα protein remained after 6 hours of exposure (Fig. 3C). It is important to emphasize that the E2-induced reduction in ERα protein expression was observed only at the highest concentration of the ligand tested (100 nM; Fig. 3D). In contrast, E2-stimulated proliferation of MCF-7 cells at only 100 pM [13]. The observed reduction in ERα protein expression after exposure to both fulvestrant and E2 did not occur when cells were pre-exposed to MG132, a wide-spectrum proteasome inhibitor [51] (Figs. 3F–G), confirming the reported proteasome-dependent nature of fulvestrant- and E2-induced degradation of ERα protein [52], [53]. Exposure to a high concentrations of MG132 (125 nM) caused increase in ERα protein expression to a level even greater than cells not exposed to fulvestrant, suggesting the presence of basal ERα protein turnover (i.e., persistent synthesis and proteasomal degradation) in MCF-7 cells.

**Figure 4:**
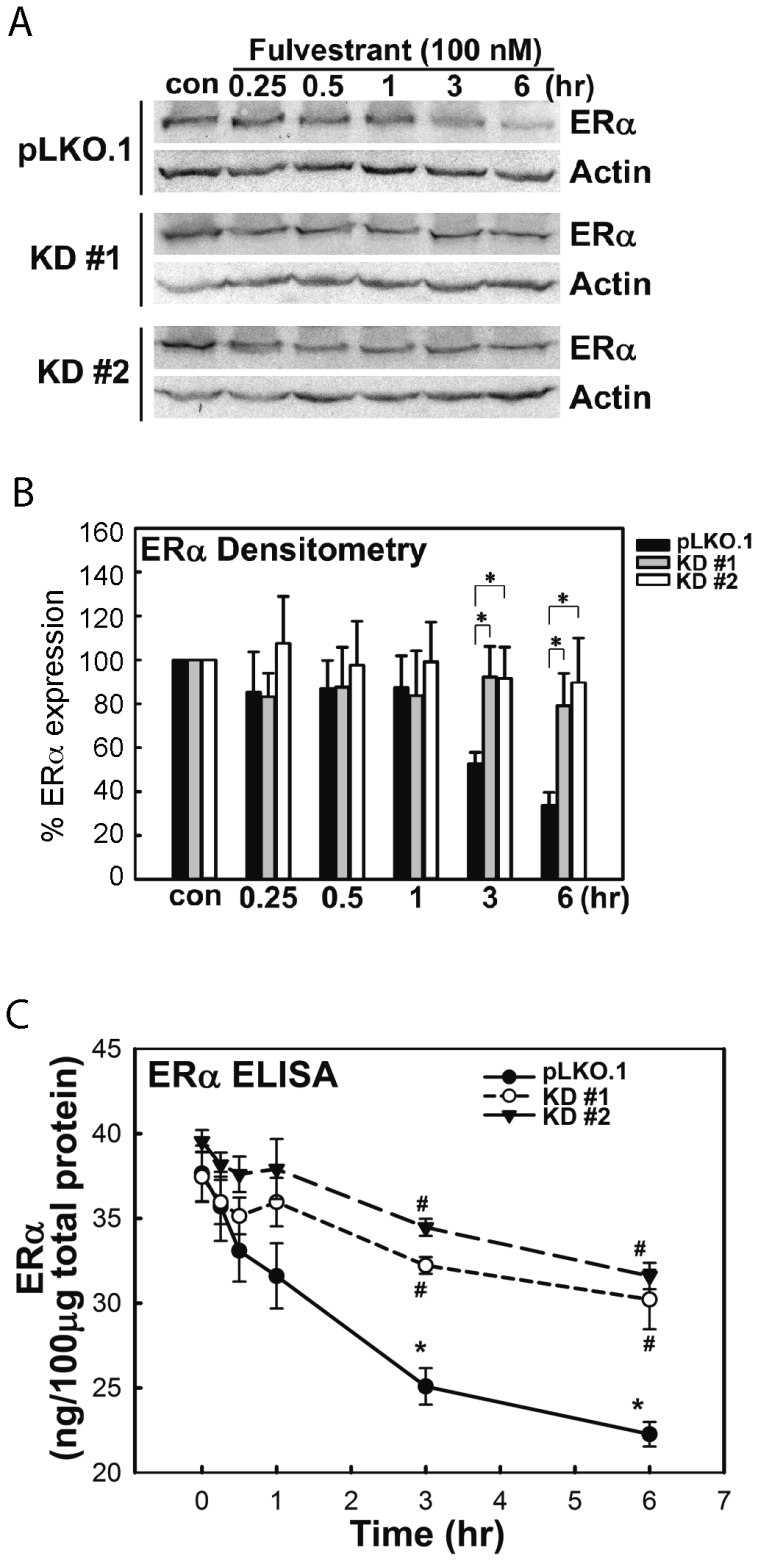
CSK is required for fulvestrant-induced ERα protein degradation in MCF-7 cells. (A, B) RNAi knockdown of CSK protein expression caused resistance of intracellular ERα protein to fulvestrant-induced degradation: Western blotting. Cells were infected with control (pLKO.1) or two CSK-knockdown shRNA lentivirus clones and subjected to exposure to fulvestrant. Expression of ERα protein was determined by Western blotting at varying time points of exposure (A). Intensities of ERα protein bands were determined by densitometry (B, mean±SEM of three independent experiments. Asterisk indicates statistical significance, p<0.05). (C) Similar experiments as shown in panels (A, B) were performed, but amounts of ERα protein in total cellular protein were determined by ELISA (mean±SEM of three independent experiments; *, p<0.05 to vehicle control; #, p<0.05 to pLKO.1-infected cells exposed to fulvestrant for the same period).

Although fulvestrant and tamoxifen are similar in inhibiting estrogen signaling, their mechanisms of actions differ. Whereas fulvestrant cause proteasomal degradation of ERα protein in breast cancer cells [11], [31], [35], tamoxifen is known to stabilize ERα protein [54], [55]. To explain the fulvestrant-specific resistance of the CSK-knockdown MCF-7 cells without affecting their tamoxifen sensitivity, we hypothesized that CSK may be required for fulvestrant-induced proteasomal degradation of ERα protein. To test this hypothesis, we examined time-dependent degradation of ERα protein after exposure to 100 nM fulvestrant in MCF-7 cells infected with pLKO.1 control or CSK shRNA lentiviruses (Fig. 4). Infection with both CSK shRNA lentiviruses #1 and #2 almost completely abolished the fulvestrant-induced ERα protein degradation when examined by Western blotting. However, infection with pLKO.1 control virus did not significantly alter the action of fulvestrant effect (Figs. 4A and 4B). To obtain more quantitative ERα protein data, we repeated this experiment but using ELISA (Fig. 4C). After exposure to fulvestrant for 6 hours, ERα protein in pLKO.1-infected control cells was reduced from 37.65±1.64 ng/100 µg total extractable cellular protein to 22.27±0.72 ng/100 µg. On the other hand, ERα expression in cells infected with CSK shRNA lentiviruses was slightly reduced from 37.45±1.48 ng/100 µg to 30.22±1.75 ng/100 µg (shRNA #1) and 39.55±0.65 ng/100 µg to 31.60±0.77 ng/100 µg (shRNA #2) (Fig. 4C). Thus, agreeing with the Western blotting data, ERα expression determined by ELISA was reduced to 33.6±6.1% of vehicle-exposed control after 6-hour exposure to 100 nM fulvestrant in pLKO.1-infected cells. In contrast, cells infected with CSK shRNA lentiviruses retained 79.08±14.72% (shRNA #1) and 89.56±20.44% (shRNA #2) ERα protein expression as compared to vehicle control at under the same conditions. When CSK protein was re-expressed in the cells infected with the CSK shRNA #1 lentivirus by transfection of an expression plasmid, the fulvestrant-induced degradation of ERα protein was partly rescued (Fig. S4). However, re-expression of CSK did not reinstate the fulvestrant-induced MCF-7 cell death (data not shown), presumably due to the transient nature of CSK re-expression from a plasmid vector. Thus, RNAi knockdown of CSK expression strongly suppresses the fulvestrant-induced ERα protein degradation in MCF-7 cells.

To determine whether the suppression of the fulvestrant-induced ERα protein degradation by RNAi knockdown of CKS is also observed in another cell culture model, we repeated the same experiment with T47D human breast cancer cells. Whereas T47D cells are dependent on estrogen for their proliferation, they survive in the absence of estrogen signaling due to the loss-of-function mutation of the p53 tumor suppressor protein [56]. Thus, when T47D cells were exposed to fulvestrant, cells neither proliferated nor died (Fig S5A). Expression of ERα protein in T47D cells infected with the pLKO.1 control lentiviral vector was strongly diminished upon exposure to 100 nM fulvestrant for 3–9 hours (Figs. S5C, S5E), reproducing the observation made with MCF-7 cells (Fig. 2). In contrast, ERα protein was significantly resistant to degradation in fulvestrant-exposed T47D cells infected with the CSK-KD#1 shRNA lentivirus (Figs. S5D, S5E), whose CSK expression was reduced by approximately 70% (data not shown). The resistance was partly reversed by re-expression of CSK from an exogenous vector (Fig. S5E). These results indicate that CSK is required for the fulvestrant-induced ERα protein degradation in T47D cells even though fulvestrant does not show significant cytocidal action in this cell line.

### Small-molecular-weight inhibitors of c-Src do not affect fulvestrant-induced MCF-7 cell death or ERα protein degradation

CSK (c-Src tyrosine kinase) is a protein tyrosine kinase that phosphorylates the C-terminal regulatory tyrosine of c-Src oncoprotein, which itself is a protein tyrosine kinase [57]. Phosphorylation by CSK suppresses the kinase activity of c-Src as well as other Src-family tyrosine kinases, and this is a physiological mechanism regulating c-Src activity both in mammals and Drosophila [57], [58]. Roles of CSK in metastasis of human cancer cells have also been suggested [58]. c-Src directly phosphorylates nuclear hormone receptors such as androgen receptor or ERα, and this phosphorylation is required for steroid hormone signaling [59]–[61]. Thus, c-Src links signaling initiated by the plasma membrane receptor tyrosine kinases such as epidermal growth factor receptor and steroid hormone signaling [62]–[64].

**Figure 5:**
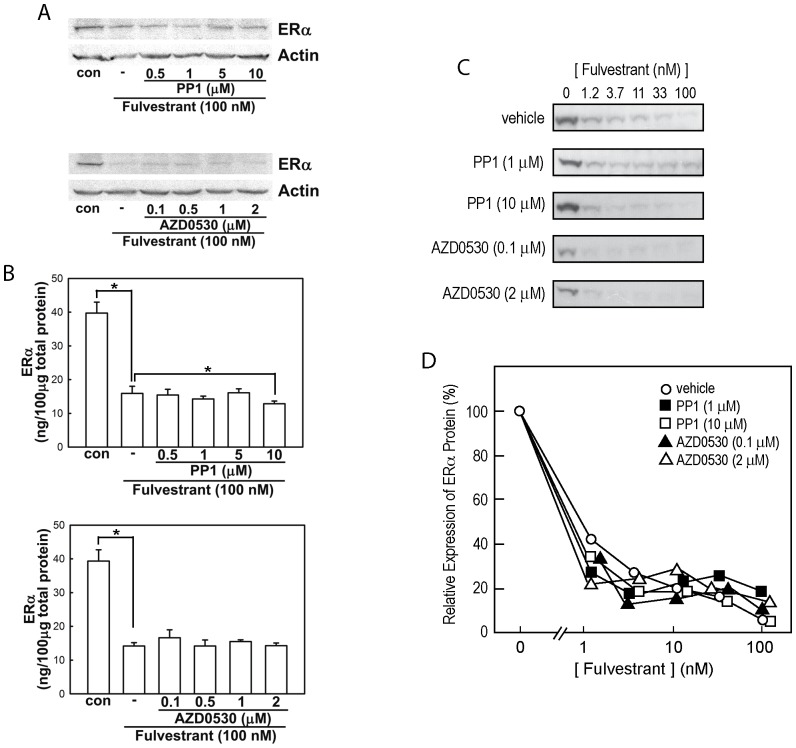
PP1 or AZD0530 tyrosine kinase inhibitors had no effect on ERα protein degradation in MCF-7 cells. In panels (**A**) and (**B**), ERα protein expression after 6-hour exposure to 100 nM fulvestrant in the presence of PP1 or AZD0530 was determined by Western blotting (A) and ELISA (B, mean±SEM of three or more independent experiments. Asterisks indicate statistical significance, p<0.05, to fulvestrant-only group). In panels (**C**) and (**D**), fulvestrant concentration was reduced as indicated, and ERα protein expression after 6-hour exposure in the presence of PP1 or AZD0530 was determined by Western blotting (**C**). Panel (**D**) shows a typical densitometric quantitation of the ERα protein band. Three independently performed experiments did not show statistically significant effects of PP1 or AZD0530.

To determine whether CSK affects fulvestrant-induced ERα protein degradation through altering c-Src kinase activity, we examined effects of small-molecular-weight inhibitors of c-Src tyrosine kinase on fulvestrant-induced MCF-7 cell death and ERα degradation. PP1 is a relatively specific inhibitor of c-Src although it also inhibits tyrosine kinase activities of c-Kit and Bcr-Abl [65]. AZD0530 (a.k.a. saracatinib) selectively inhibits c-Src and Bcr-Abl kinases [66]–[69]. We reasoned that, if CSK is required for fulvestrant-induced cell death or ERα protein degradation through suppression of c-Src, inhibition of c-Src tyrosine kinase by chemical inhibitors would pharmacologically mimic CSK activation and show the opposite effect of CSK knockdown-namely, enhanced MCF-7 cell sensitivity to fulvestrant actions. However, by our hands, neither PP1 (0.5–10 µM) nor AZD0530 (0.1–2 µM) significantly affected the fulvestrant-induced MCF-7 cell death (Fig. S6). These c-Src inhibitors did not affect the fulvestrant-induced ERα protein degradation, either (Fig. 5). Repeated experiments with reduced fulvestrant concentrations or shorter exposure times did not reveal any effects of PP1 or AZD0530 (data not shown). Effective inhibition of c-Src tyrosine kinase activity by these compounds was confirmed by strong suppression of epidermal growth factor-induced phosphorylation of Tyr416, a well-accepted hallmark of c-Src activation [58], [70]–[73] (Fig. S7). Interestingly, c-Src kinase activity was not significantly enhanced in the MCF-7 cells whose CSK expression was suppressed by RNAi knockdown (Fig. S7C), suggesting that c-Src regulation by CSK may have been replaced by other mechanisms.

## Discussion

Activation of ERα by E2 triggers assembly of an active transcription complex, which in turn signals polyubiquitination and proteasomal degradation of the liganded ERα protein [44], [74]–[80]. Chu *et al.* reported that the E2-triggered proteasomal degradation of ERα protein in MCF-7 cells were enhanced by activation of c-Src [81]. Binding of fulvestrant to ERα also causes proteasomal degradation although it is not associated with transcriptional activation. Because the fulvestrant-triggered ERα protein degradation is 10 times faster than that triggered by E2 in MCF-7 cells [82], mechanisms of the ERα protein degradation invoked by these two ligands may significantly differ. Our present study provided evidence that CSK, the negative regulator protein tyrosine kinase of c-Src, is required for fulvestrant-triggered ERα protein degradation in MCF-7 cells, which appears to be opposite to the report of Chu *et al.* [81]. However, the apparent lack of c-Src activation in the MCF-7 cells whose CSK expression was stably suppressed by RNAi knockdown (Fig. S7) may suggest that c-Src might be regulated by other mechanisms in the absence of CSK in these cells. Rengifo-Cam *et al.* demonstrated activation of c-Src by 48-hour adenoviral overexpression of a dominant-negative CSK in human colorectal cancer cells [58]. Since our present study was performed using stable CSK-knockdown cultures of MCF-7 cells, transient activation of c-Src, if any, could have been suppressed by compensating mechanisms. Our attempts to suppress the intracellular CSK actions by dominant-negative CSK as reported by Rengifo-Cam *et al.* were unsuccessful due to nonspecific induction of apoptosis of MCF-7 cells, which express wild type p53 tumor suppressor protein as the majority of human ER+/PR+/HER2- breast cancers [56], [83].

In MCF-7 cells, fulvestrant mobilizes ERα into the nuclear matrix in a manner dependent on interactions between the helix 12 domain of ERα and cytokeratins 8 or 18 [75], [84]–[86]. Mobilization of ERα to nuclear matrix is necessary for polyubiquitination of ERα protein by a mechanism involving the NEDD8 ubiquitin-like protein and the Uba3-containing NEDD8-activating enzyme [87] and subsequent degradation by the 26S proteasome [85]. Using a panel of kinase inhibitor/activator chemicals, Marsaud et al. observed that protein kinase C is an enhancer of the fulvestrant-induced proteasomal ERα degradation in MCF-7 cells whereas protein kinase A, MAPKs, and phosphatidyl-inositol-3-kinase act as suppressors [82]. Tsai et al. also reported that forskolin, a potent activator of protein kinase A, prevents fulvestrant-induced ERα protein degradation in MCF-7 cells [88]. Thus, the signaling involving protein kinases seems to have significant roles in regulating the fulvestrant-induced proteasomal ERα protein degradation in breast cancer cells. Our finding that CSK is required for this fulvestrant action provides additional insights into how the kinase/phosphatase-mediated intracellular signaling network in human breast cancer cells is closely linked to antiestrogen sensitivity.

A number of previous studies including ours [35] isolated fulvestrant-resistant variants of MCF-7 cells after long-term exposure of the polyclonal MCF-7 cell culture to fulvestrant. These studies agree that the fulvestrant resistant variants isolated with this approach did not depend on estrogen signaling because other signaling pathways (e.g., EGF receptor, ERK1/2, c-Met, and AKT [89]–[92]) supported their proliferation and survival. In these fulvestrant resistant variants, the fulvestrant-induced ERα protein degradation was intact. By siRNA transfection-based RNAi knockdown screenings generating synthetic resistance to tamoxifen, Iorns et al. identified CDK10, CRK7, and MAP2K7 as kinases necessary for tamoxifen sensitivity of MCF-7 cells [30]. Again, knockdown of any of these three kinases caused estrogen insensitivity in MCF-7 cells. Our shRNA lentivirus-based RNAi knockdown screenings generating synthetic resistance to fulvestrant identified MAP2K7 and CSK as kinases necessary for fulvestrant-induced MCF-7 cell death. Independent identification of MAP2K7 as a kinase required for sensitivities of both tamoxifen (Iorns et al. [30]) and fulvestrant (our present study) supports validity of the RNAi knockdown screenings performed in our present study. Since MAP2K7 knockdown did not affect the fulvestrant-induced proteasomal degradation of ERα protein (data not shown), CSK is a unique protein whose knockdown in MCF-7 cells does not cause estrogen insensitivity but leads to drug resistance due to cancellation of the induced ERα protein degradation.

Details of the link between CSK knockdown and cancellation of the fulvestrant-induced proteasomal ERα degradation remain to be determined. Attempts made in our present study did not establish roles of c-Src in the requirement of CSK for the fulvestrant-induced ERα protein degradation although the possible involvement of c-Src in this mechanism cannot be denied. As CSK directly phosphorylates not only c-Src but also the transcription factor [93] and the ATP-activated P2X_3_ receptor [94], these non-Src CSK substrates might also be involved in the fulvestrant-induced ERα protein degradation. In this context, it is interesting that phosphorylation of c-Jun at Tyr26 and Tyr170 by CSK causes ubiquitination and proteasomal degradation of the c-Jun protein [93].

In summary, our present study identified CSK as a novel protein tyrosine kinase required for the fulvestrant-induced proteasomal degradation of ERα protein in MCF-7 cells. RNAi knockdown of CSK caused specific resistance to fulvestrant without affecting MCF-7 cell sensitivities to tamoxifen or paclitaxel, suggesting possible importance of CSK for better understanding of the mechanisms of the cytocidal action of fulvestrant in human breast cancer cells.

## Materials and Methods

### Chemicals

Fulvestrant (Faslodex^®^/ICI 182,780; research-grade pure chemical) and PP1 were purchased from Tocris (Ellisville, MO). Crystal violet, 4-Hydroxytamoxifen, paclitaxel, and MG132 were from Sigma (St. Louis, MO). Puromycin hydrochloride and 17α-Estradiol was from Calbiochem (Gibbstown, NJ). AZD0530 was obtained from Selleck Chemicals Co. (ShangHai, China). Recombinant human epidermal growth factor (EGF) was purchased from R&D Systems (Minneapolis, MN).

### Cell Culture

MCF-7 human breast cancer cell culture (BUS stock) was provided by C. Sonnenschein and A. M. Soto (Tufts University) [95], [96], and its fulvestrant-sensitive monoclonal subline (W2) was described in our recent study [35]. Our present study was performed using the W2 clone of MCF-7 cells. T47D human breast cancer cells were purchased from ATCC (Manassas, VA). All cells were maintained in Dulbecco's MEM (DMEM) supplemented with 5% FCS (HyClone, DEFINED grade; Thermo Scientific, Waltham, MA) in 10% CO_2_ at 37 °C. To examine ERα protein degradation induced by 17α-estradiol, subconfluent cells were washed three times with phenol red-free DMEM (containing no serum) and incubated in the last wash medium for 60 minutes at 37 °C. Medium was then replaced by phenol red-free DMEM supplemented with 5% charcoal/dextran-stripped FCS (HyClone) and hormone-starved for another 24 hours before exposure to 17α-estradiol [13].

### shRNA Lentivirus Production and Infection

Lentiviruses expressing shRNA species targeting specific human mRNA transcripts were produced using the pLKO.1 vector harboring the puromycin-resistance marker following published protocols [33]. Subconfluent HEK293T packaging cells growth in 96-well plates were transfected with arrayed, pLKO.1-based shRNA expression plasmids for human kinome screening (6,560 protein kinases and phosphatases) obtained from The RNAi Consortium (Broad Institute, Cambridge, MA) with the expression plasmids for VSV-G surface antigen and the core lentiviral genome. For infection, 5 × 10^3^ cells were seeded into wells of 96-well plate and allowed to attach for 24 hours. Cells (5∼10 × 10^4^ cells/well)were infected with lentiviruses (4 × 10^4^ IU; MOI = 4∼8) in the presence of 8 µg/ml polybrene under 1,200 x g gravity by spinning for 60 minutes. Medium was changed 48 hours after infection, and successful infected cells were selected by puromycin (2.5 µg/ml) for 48 hours.

### Cell Viability and Crystal Violet Staining

Cell viability was assessed by crystal violet staining. Cells grown in 96-well plate were washed with PBS twice and then fixed with 12% formaldehyde. After 10 minutes incubation at room temperature, cells were completely dried and stained with 1% crystal violet for 5 minutes. Stained cells were washed with tap water and subjected to spectrophotometric quantitation (OD 590 nm) using SpectraMax M5 (Molecular Devices, Sunnyvale, CA).

### Protein Analyses

Western blotting was performed as we previously described [97]. Briefly, cells were washed with ice cold PBS and lysed with a RIPA buffer (150 mM NaCl, 25 mM Tris HCl pH 7.6, 1% NP-40, 1% sodium deoxycholate, 0.1% SDS). Protein concentration was determined by bicinchoninic acid (BCA) protein assay kit (Pierce, Rockford, IL) with BSA as a standard. 80 µg of total cellular protein was separated on 7.5% Tris-HCl polyacrylamide gels and transferred to PVDF membranes (Bio-Rad, Richmond, CA). The membranes were incubated for 1 h with 5% dry skim milk in PBST buffer (PBS containing 0.05% tween 20) to block nonspecific binding and then incubated with primary antibodies (x1000 dilution) overnight at 4 °C. The primary antibodies were: anti-human actin (goat IgG, sc.-1616/I-19, Santa Cruz Biotechnology, Santa Cruz, CA), anti-human ERα (rabbit IgG, sc-542/MC-20 and sc-544/G-20, Santa Cruz Biotechnology), and anti-human CSK (goat IgG, ab744–100, Abcam, Cambridge, MA). The membranes were washed with PBST and then incubated with peroxidase-conjugated secondary antibodies (donkey anti-goat IgG or goat anti-rabbit IgG, x3000 dilution, Santa Cruz Biotechnology) for 1 h at room temperature. All antibodies were diluted in 1% dry skim milk in PBST buffer. Protein bands were visualized by enhanced chemiluminescence (GE Healthcare, Piscataway, NJ) using Kodak BioMax MR films (Perkin Elmer, Waltham, MA). Signal intensities of protein bands were quantitated by densitometry from at least three independent experiments using ImageQuant system (GE Healthcare).

Phosphorylation of c-Src was examined using the Odyssey infrared imaging system (LI-COR Biosciences, Lincoln, Nebraska) as previously described [98] using rabbit anti-phosphorylated human c-Src polyclonal antibody (P-Tyr416, #2101, Cell Signaling Technology, Danvers, MA) and mouse anti-human c-Src monoclonal antibody (IgG_1_, sc.-32789, Santa Cruz Biotechnology) as primary antibodies. Secondary antibodies (IRDye 680 donkey anti-rabbit IgG and IRDye 800 donkey anti-mouse IgG) were purchased from LI-COR Biosciences. For c-Src kinase activity assay, c-Src protein was immunoprecipitated using the anti-human c-Src monoclonal antibody and protein G beads and subjected to the ProFluor Src family kinase assay (Promega, Madison, WI) following the manufacturer's instructions.

### ERα ELISA

Cell lysates were prepared with the RIPA buffer, and 100 µg of total protein was subjected to ERα ELISA (Active Motif; Carlsbad, CA) following manufacturer's instructions. Absorbance at 450 nm was determined by Synergy HT plate reader (BioTek, Winooski, VT).

### Expression of CSK by transient transfection of an expression plasmid

An expression plasmid for human full-length CSK (Cat. # RC210758) and its control vector (pCMV6-ENTRY) was purchased from OriGene Technologies (Rockville, MD). The plasmid expressed an open reading frame for human CSK transcript variant 1 tagged C-terminally with the myc and DDK epitope peptides and placed under the CMV promoter. Subconfluent cells were transfected with the CSK expression plasmid or the control plasmid together with an expression plasmid for a green fluorescence protein (S65T red shift mutant of EGFP) using TransIT-LT1 transfection reagent following the manufacturer's instructions (Mirus Bio, Madison, WI). High transfection efficiency (>70%) was confirmed by expression of the EGFP observed using a fluorescence microscope.

### Statistics

Values are expressed as mean±SEM of at least three independent experiments. One-way analysis of variance (ANOVA) was performed on the values followed by Tukey post-hoc test in GraphPad PRISM6 statistic software package (GraphPad Software, La Jolla, CA).

## Supporting Information

Figure S1 **RNAi knockdown of CSK in MCF-7 cells causes resistance to fulvestrant: Crystal violet staining data.** Cells were infected with empty lentivirus vector (pLKO.1) or two independent clones of lentiviruses expressing different shRNA species targeting CSK (CSK KD #1 and #2) and exposed to puromycin, fulvestrant, or vehicle for 7 days.
(PDF)

Figure S2 **RNAi knockdown of CSK in MCF-7 cells and resistance to fulvestrant.** (**A**) Cells were infected with empty lentivirus vector (pLKO.1) or lentivirus clones expressing different shRNA species targeting CSK as listed in Table S1 and subjected to Western blotting quantitation of CSK protein expression. CSK-KD, CSK knockdown. (**B**, **C**) Fulvestrant resistance of MCF-7 cells infected with shRNA lentiviruses targeting CSK. Cells infected with shRNA lentivirues were exposed to 100 nM fulvestrant or vehicle for 7 days. (**B**) Gross appearance of cell culture after crystal violet staining. (**C**) Phase contrast microscopic images. MCF-7 cells expressing CSK (MCF-7 W2 and pLKO.1 infected cells) showed massive apoptotic death after fulvestrant exposure whereas cells subjected to RNAi knockdown of CSK survived. MCF-7 cells with CSK knockdown often showed significant pileup growth appearance as shown in this picture.
(PDF)

Figure S3 **RNAi knockdown of CSK does not affect MCF-7 cell sensitivity to tamoxifen or paclitaxel.** Cells were infected with empty lentivirus vector (pLKO.1) or two independent clones of lentiviruses expressing different shRNA species targeting CSK (CSK KD#1 and #2) and then exposed to 1 µM 4-hydroxytamoxifen (4-OHT) for 10 days (A) or 1–1000 nM paclitaxel for 2 days (B). Cell viability was determined by crystal violet staining. Quantified data obtained by spectrophotometry of the stained cells are shown in Fig. 2.
(PDF)

Figure S4 **Re-expression of CSK in MCF-7 cells rescues fulvestrant-induced ERα protein degradation.** (**A**) Diminished CSK protein expression in MCF-7 cells subjected to lentiviral RNAi knockdown and re-expression by transfection of a CSK expression plasmid: Western blotting. MCF-7 cells were infected with pLKO.1 control lentivirus (lane 1) or the CSK-KD#1 shRNA lentivirus (lanes 2, 3). The cells infected with the CSK-KD#1 virus were further subjected to transfection of an expression plasmid for human CSK (lane 3) or a control plasmid harboring no insert (lane 2). Expression of CSK protein was determined by Western blotting 24 hours after transfection. (**B**) Time-course of ERα protein expression in MCF-7 cells exposed to fulvestrant: Western blotting. Intensities of ERα protein bands were determined by densitometry (C, mean ± SEM of three independent experiments. *Asterisk* indicates statistical significance (p<0.05) against the control without exposure to fulvestrant (con). *Sharp* indicates statistical significance (p<0.05) between CSK knockdown cells with or without re-expression of CSK1 from a plasmid.
(PDF)

Figure S5 **Re-expression of CSK in MCF-7 cells rescues fulvestrant-induced ERα protein degradation.** (**A**, **B**) Effects of E2 and fulvestrant on proliferation and survival of T47D cells. Cells were for up to 6 days (**A**) or 11 days (**B**) in the presence or absence of E2 and/or fulvestrant in the medium, and the live cell numbers in the culture were determined by crystal violet staining. Note that live cell number was not decreased in the presence of fulvestrant even though cells were not proliferated in this condition, either. (**C**–**E**) Changes in ERα protein expression in T47D cells exposed to fulvestrant. T47D cells infected with pLKO.1 control lentivirus (**C**) or the CSK-KD#1 shRNA lentivirus targeting CSK (**D**) were exposed to 100 nM fulvestrant or vehicle (ethanol) for 3, 6, or 9 hours (control, no exposure) and then subjected to Western blotting determination of ERα protein expression. Intensities of ERα protein bands were determined by densitometry (E, mean ± SEM of three independent experiments. Asterisk indicates statistical significance (p<0.05) against control; sharp indicates significant differences between the pLKO.1-infected and the CSK-KD#1 infected cells observed when cells were exposed to fulvestrant (p<0.05, *t*-test).
(PDF)

Figure S6 **PP1 or AZD0530 tyrosine kinase inhibitors had no effect on fulvestrant-induced cell death.** Cells were exposed to PP1 (0.1–2 µM, A) or AZD0530 (0.1–2 µM, B) for 30 minutes and then exposed to 100 nM fulvestrant in the presence of the same c-Src kinase inhibitors for 5 days. Cell viability was determined by crystal violet staining. Representative crystal violet staining images are shown. Amounts of stained cells were determined by spectrometry as shown in the bar graphs (mean ± SEM of three independent experiments; asterisk indicates statistical significance p<0.05 against the vehicle control, sharp indicates significance against the absence of AZD0530).
(PDF)

Figure S7 **c-Src phosphorylation and kinase activity in MCF-7 cells.** (**A**) Fulvestrant does not induce c-Src phosphorylation. MCF-7 cells were exposed to 100 nM fulvestrant or 20 ng/ml EGF for 5, 10, and 30 minutes and subjected to Western blotting of Tyr416-phosphorylated and total c-Src. (**B**) Inhibition of EGF-induced c-Src Tyr-416 phosphorylation by PP1 or AZD0530. Cells were exposed to 10 µM PP1 or 2 µM AZT for 30 min and then stimulated with 20 ng/ml EGF for another 30 minutes. Phosphorylation of c-Src at tyr416 was determined by Western blotting. Typical images of three repeated experiments are shown for panels (**A**) and (**B**). Asterisks indicate statistical significance (p<0.05). (**C**) c-Src kinase activities in MCF-7 cells. c-Src was enriched by immunoprecipitation and subjected to kinase assay (mean ± SEM of three experiments). 1–3, MCF-7 cells infected with pLKO.1 control lentivirus exposed to vehicle (1), 10 µM PP1 (2), or 2 µM AZT (3) for 30 min. 4–5, MCF-7 cells infected with CSK-KD#1 (4) or CSK-KD#2 (5) shRNA lentiviruses.
(PDF)

Table S1 **The TRC collection of shRNA lentiviral clones targeting human CSK.**
(PDF)
