# Supplementary figures and images for: Fulvestrant-Induced Cell Death and Proteasomal Degradation of Estrogen Receptor α Protein in MCF-7 Cells Require the CSK c-Src Tyrosine Kinase
Source: PLoS One. 2013 Apr 4;8(4):e60889. doi: 10.1371/journal.pone.0060889 (PMC3617152; doi:10.1371/journal.pone.0060889)

# CSK Knockdown

mock

pLKO.1

#1

#2

**Puromycin  
(2.5  $\mu$ g/ml)**

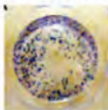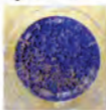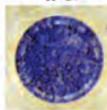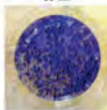

**vehicle  
(0.1% EtOH)**

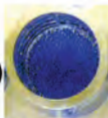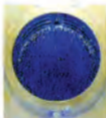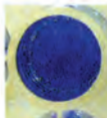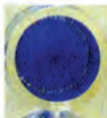

**Fulvestrant  
(100 nM)**

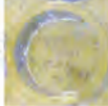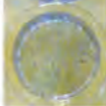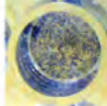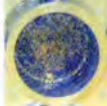

Supplement: Figure S1 — RNAi knockdown of CSK in MCF-7 cells causes resistance to fulvestrant: Crystal violet staining data. Cells were infected with empty lentivirus vector (pLKO.1) or two independent clones of lentiviruses expressing different shRNA species targeting CSK (CSK KD #1 and #2) and exposed to puromycin, fulvestrant, or vehicle for 7 days. (PDF) [file pone.0060889.s001.pdf]

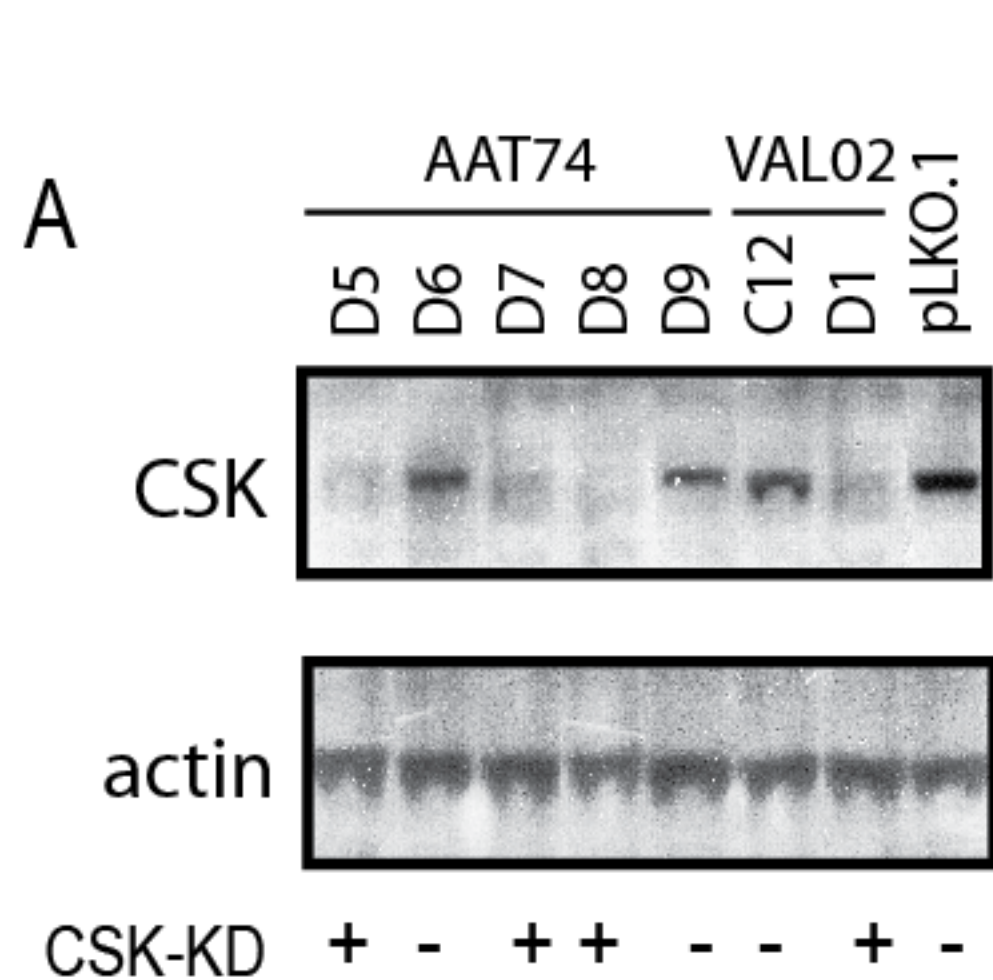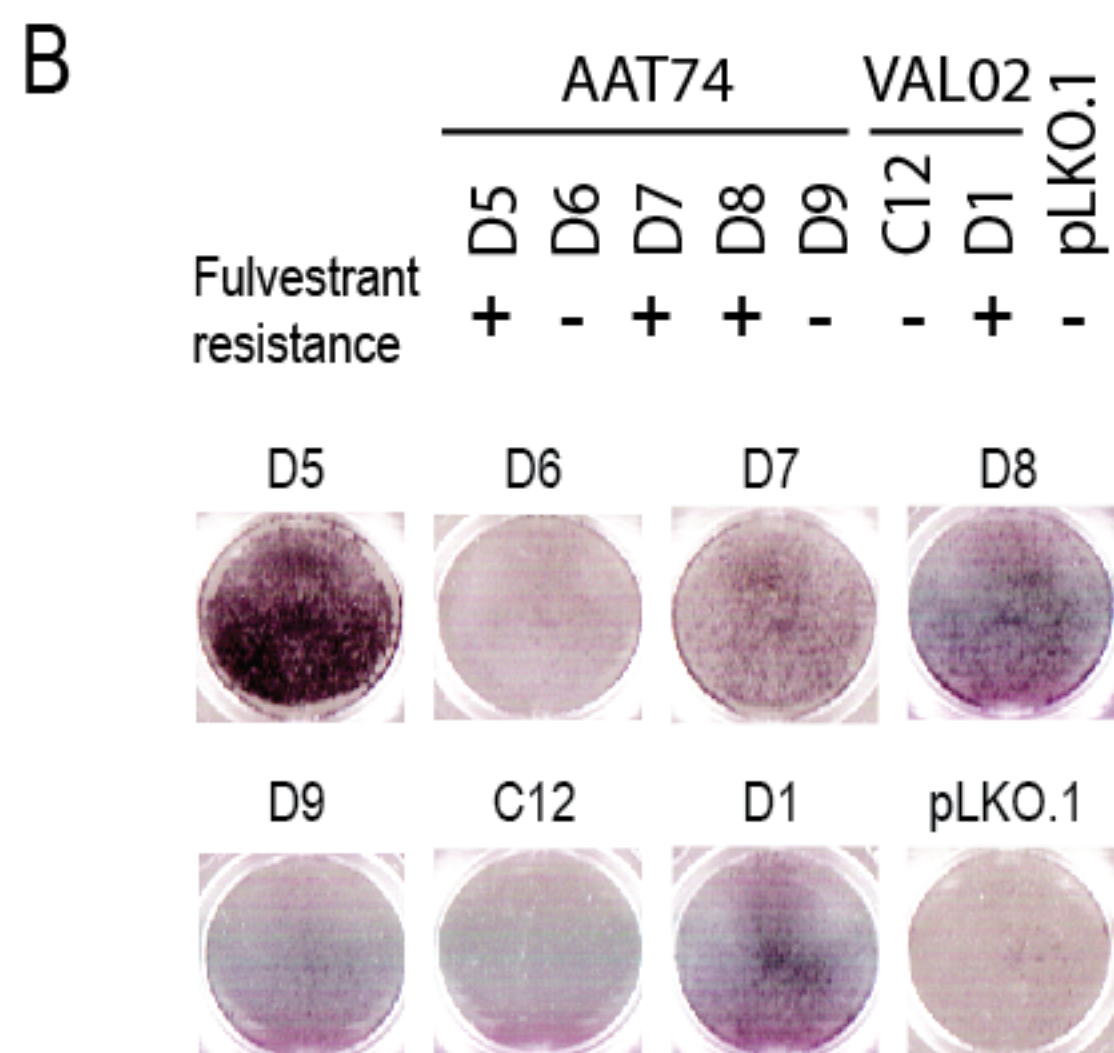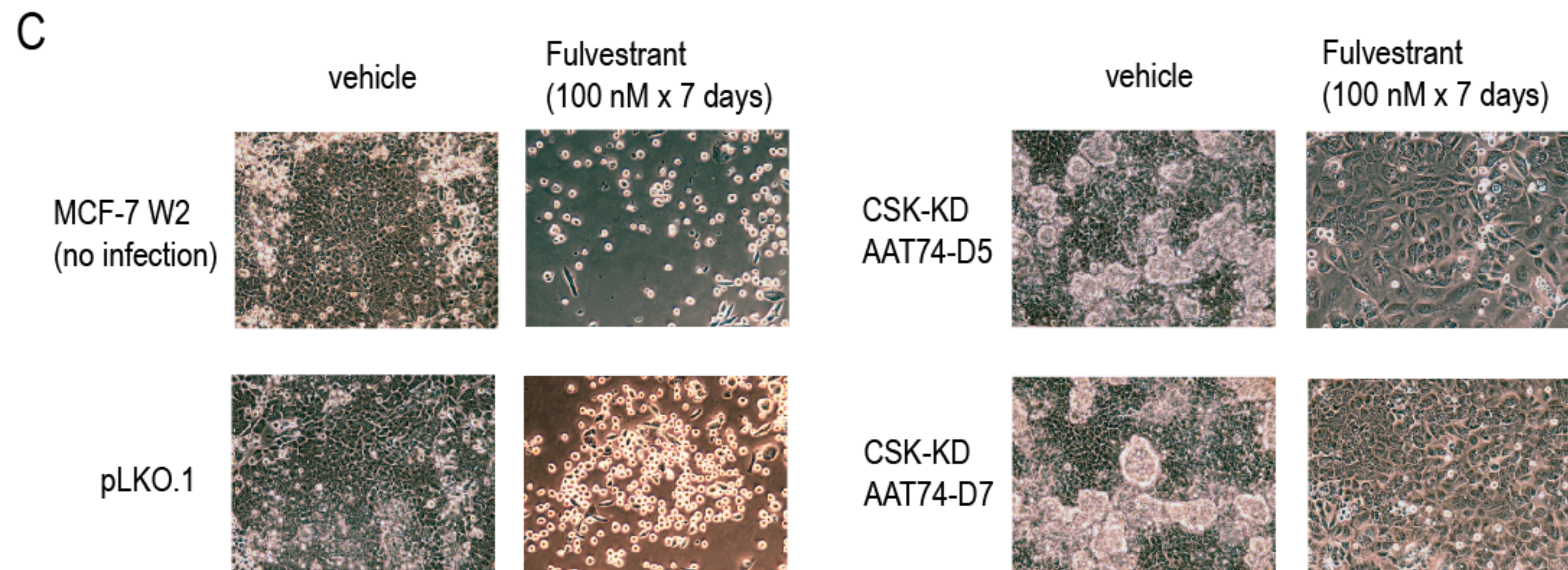

Supplement: Figure S2 — RNAi knockdown of CSK in MCF-7 cells and resistance to fulvestrant. (A) Cells were infected with empty lentivirus vector (pLKO.1) or lentivirus clones expressing different shRNA species targeting CSK as listed in Table S1 and subjected to Western blotting quantitation of CSK protein expression. CSK-KD, CSK knockdown. (B, C) Fulvestrant resistance of MCF-7 cells infected with shRNA lentiviruses targeting CSK. Cells infected with shRNA lentivirues were exposed to 100 nM fulvestrant or vehicle for 7 days. (B) Gross appearance of cell culture after crystal violet staining. (C) Phase contrast microscopic images. MCF-7 cells expressing CSK (MCF-7 W2 and pLKO.1 infected cells) showed massive apoptotic death after fulvestrant exposure whereas cells subjected to RNAi knockdown of CSK survived. MCF-7 cells with CSK knockdown often showed significant pileup growth appearance as shown in this picture. (PDF) [file pone.0060889.s002.pdf]

A

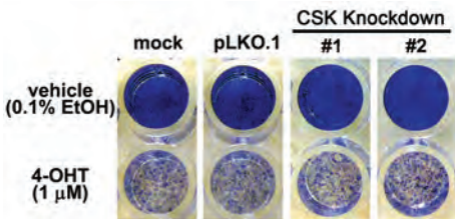

B

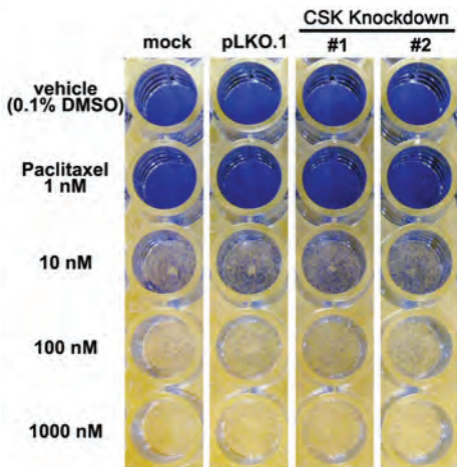

Supplement: Figure S3 — RNAi knockdown of CSK does not affect MCF-7 cell sensitivity to tamoxifen or paclitaxel. Cells were infected with empty lentivirus vector (pLKO.1) or two independent clones of lentiviruses expressing different shRNA species targeting CSK (CSK KD#1 and #2) and then exposed to 1 µM 4-hydroxytamoxifen (4-OHT) for 10 days (A) or 1–1000 nM paclitaxel for 2 days (B). Cell viability was determined by crystal violet staining. Quantified data obtained by spectrophotometry of the stained cells are shown in Fig. 2. (PDF) [file pone.0060889.s003.pdf]

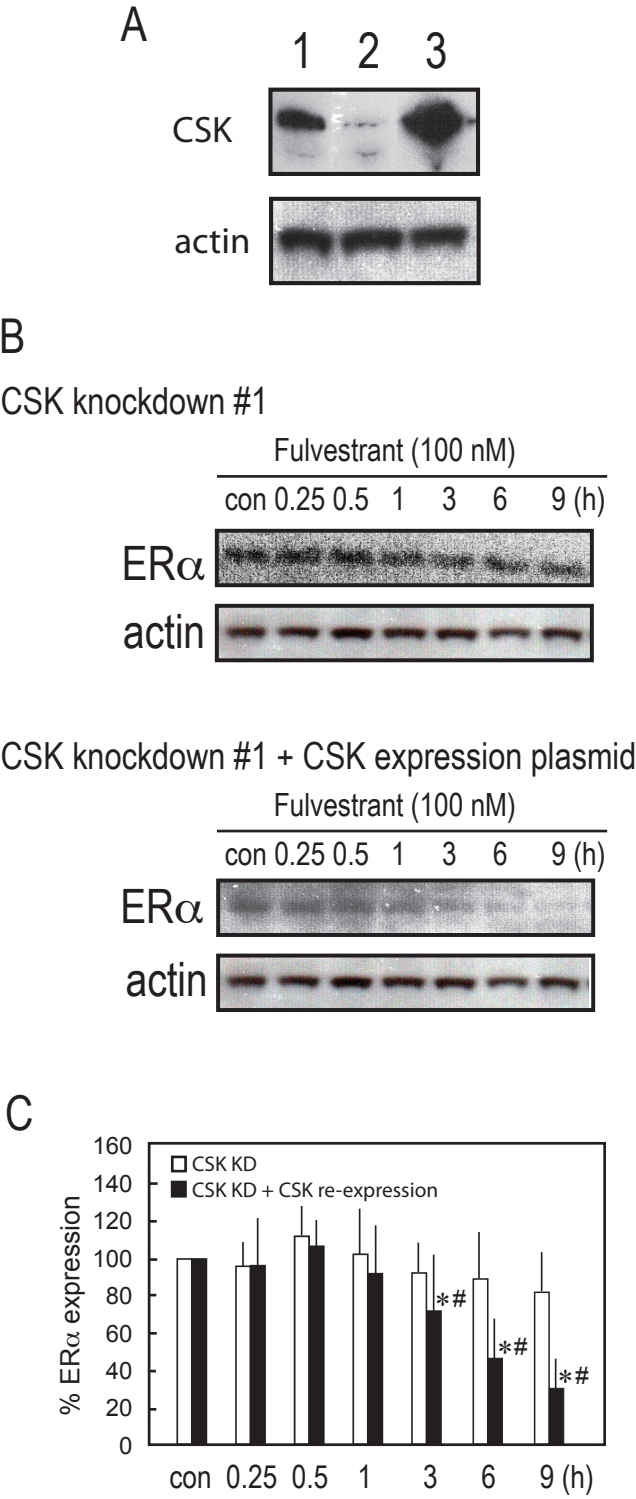

Supplement: Figure S4 — Re-expression of CSK in MCF-7 cells rescues fulvestrant-induced ERα protein degradation. (A) Diminished CSK protein expression in MCF-7 cells subjected to lentiviral RNAi knockdown and re-expression by transfection of a CSK expression plasmid: Western blotting. MCF-7 cells were infected with pLKO.1 control lentivirus (lane 1) or the CSK-KD#1 shRNA lentivirus (lanes 2, 3). The cells infected with the CSK-KD#1 virus were further subjected to transfection of an expression plasmid for human CSK (lane 3) or a control plasmid harboring no insert (lane 2). Expression of CSK protein was determined by Western blotting 24 hours after transfection. (B) Time-course of ERα protein expression in MCF-7 cells exposed to fulvestrant: Western blotting. Intensities of ERα protein bands were determined by densitometry (C, mean ± SEM of three independent experiments. Asterisk indicates statistical significance (p<0.05) against the control without exposure to fulvestrant (con). Sharp indicates statistical significance (p<0.05) between CSK knockdown cells with or without re-expression of CSK1 from a plasmid. (PDF) [file pone.0060889.s004.pdf]

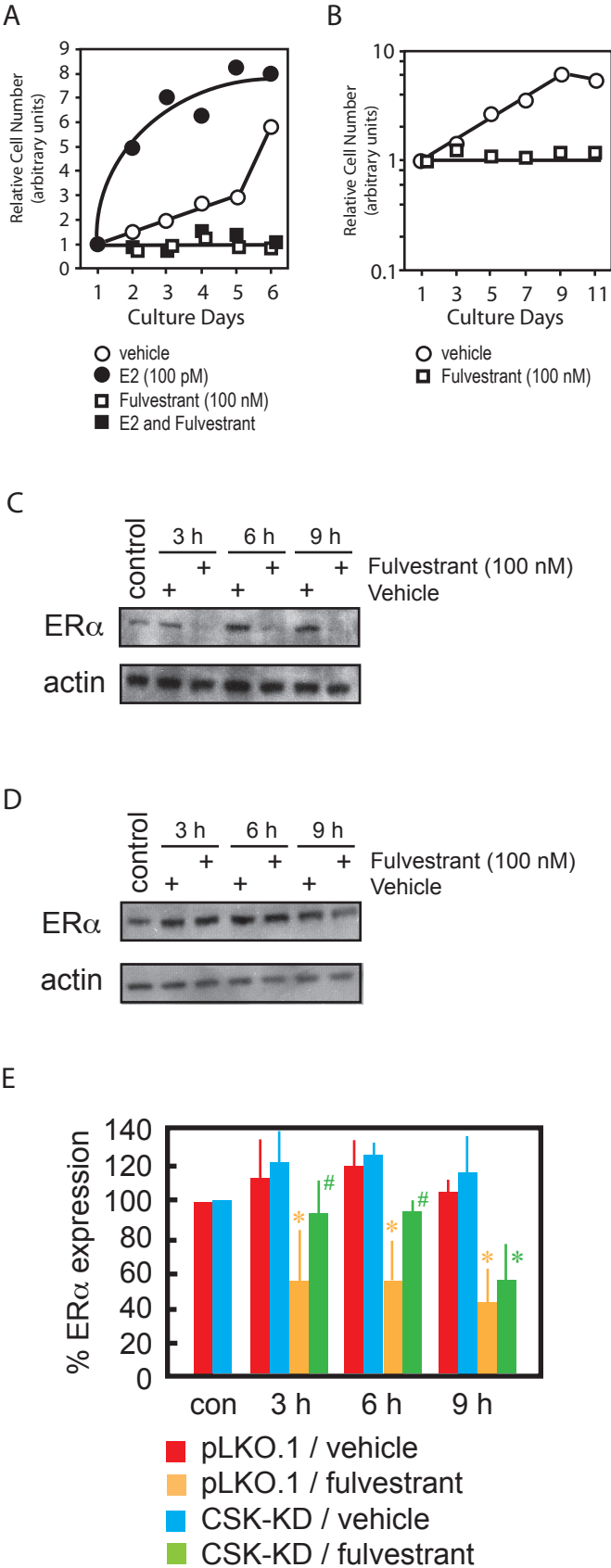

Supplement: Figure S5 — Re-expression of CSK in MCF-7 cells rescues fulvestrant-induced ERα protein degradation. (A, B) Effects of E2 and fulvestrant on proliferation and survival of T47D cells. Cells were for up to 6 days (A) or 11 days (B) in the presence or absence of E2 and/or fulvestrant in the medium, and the live cell numbers in the culture were determined by crystal violet staining. Note that live cell number was not decreased in the presence of fulvestrant even though cells were not proliferated in this condition, either. (C–E) Changes in ERα protein expression in T47D cells exposed to fulvestrant. T47D cells infected with pLKO.1 control lentivirus (C) or the CSK-KD#1 shRNA lentivirus targeting CSK (D) were exposed to 100 nM fulvestrant or vehicle (ethanol) for 3, 6, or 9 hours (control, no exposure) and then subjected to Western blotting determination of ERα protein expression. Intensities of ERα protein bands were determined by densitometry (E, mean ± SEM of three independent experiments. Asterisk indicates statistical significance (p<0.05) against control; sharp indicates significant differences between the pLKO.1-infected and the CSK-KD#1 infected cells observed when cells were exposed to fulvestrant (p<0.05, t-test). (PDF) [file pone.0060889.s005.pdf]

A

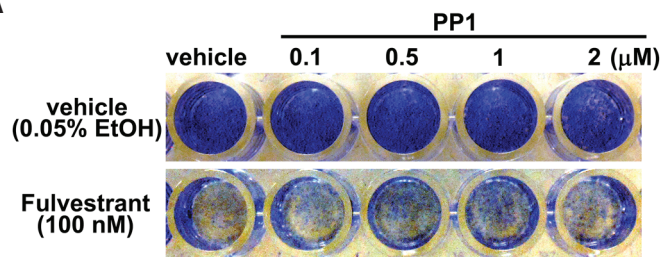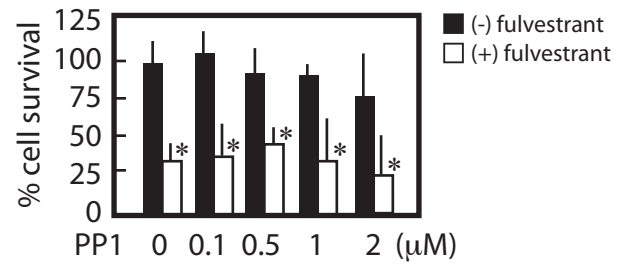

B

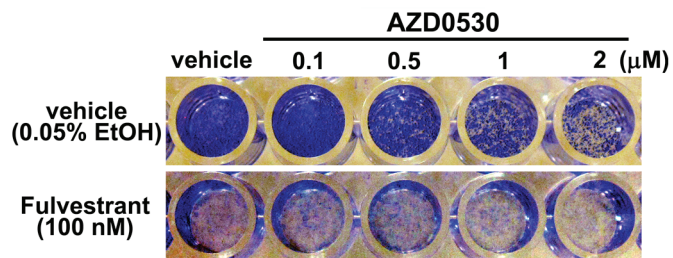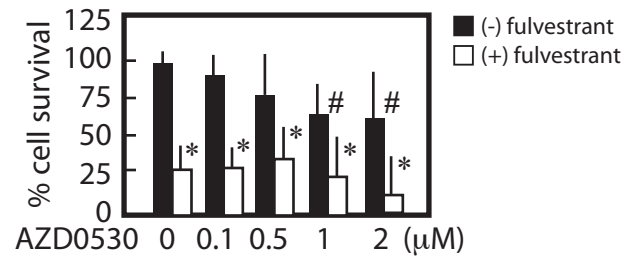

Supplement: Figure S6 — PP1 or AZD0530 tyrosine kinase inhibitors had no effect on fulvestrant-induced cell death. Cells were exposed to PP1 (0.1–2 µM, A) or AZD0530 (0.1–2 µM, B) for 30 minutes and then exposed to 100 nM fulvestrant in the presence of the same c-Src kinase inhibitors for 5 days. Cell viability was determined by crystal violet staining. Representative crystal violet staining images are shown. Amounts of stained cells were determined by spectrometry as shown in the bar graphs (mean ± SEM of three independent experiments; asterisk indicates statistical significance p<0.05 against the vehicle control, sharp indicates significance against the absence of AZD0530). (PDF) [file pone.0060889.s006.pdf]

Yeh et al. 2012  
Figure S7

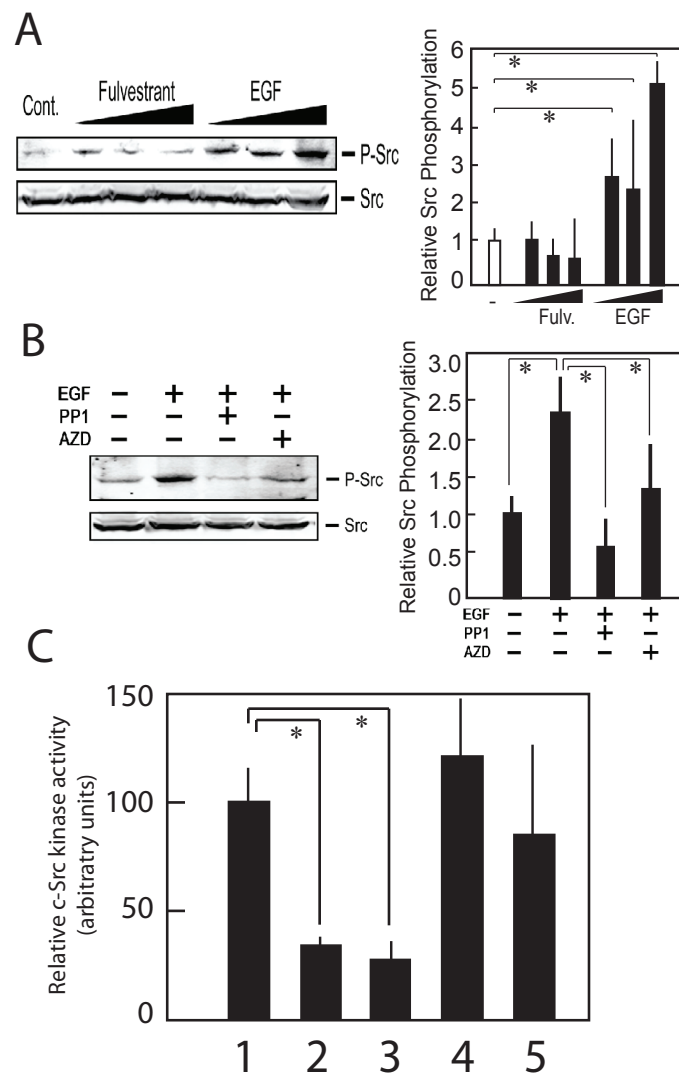

Supplement: Figure S7 — c-Src phosphorylation and kinase activity in MCF-7 cells. (A) Fulvestrant does not induce c-Src phosphorylation. MCF-7 cells were exposed to 100 nM fulvestrant or 20 ng/ml EGF for 5, 10, and 30 minutes and subjected to Western blotting of Tyr416-phosphorylated and total c-Src. (B) Inhibition of EGF-induced c-Src Tyr-416 phosphorylation by PP1 or AZD0530. Cells were exposed to 10 µM PP1 or 2 µM AZT for 30 min and then stimulated with 20 ng/ml EGF for another 30 minutes. Phosphorylation of c-Src at tyr416 was determined by Western blotting. Typical images of three repeated experiments are shown for panels (A) and (B). Asterisks indicate statistical significance (p<0.05). (C) c-Src kinase activities in MCF-7 cells. c-Src was enriched by immunoprecipitation and subjected to kinase assay (mean ± SEM of three experiments). 1–3, MCF-7 cells infected with pLKO.1 control lentivirus exposed to vehicle (1), 10 µM PP1 (2), or 2 µM AZT (3) for 30 min. 4–5, MCF-7 cells infected with CSK-KD#1 (4) or CSK-KD#2 (5) shRNA lentiviruses. (PDF) [file pone.0060889.s007.pdf]
