# Supplementary material for: Fulvestrant-Induced Cell Death and Proteasomal Degradation of Estrogen Receptor α Protein in MCF-7 Cells Require the CSK c-Src Tyrosine Kinase
Source: PLoS One. 2013 Apr 4;8(4):e60889. doi: 10.1371/journal.pone.0060889 (PMC3617152; doi:10.1371/journal.pone.0060889)
Supplement: Table S1 — The TRC collection of shRNA lentiviral clones targeting human CSK. (PDF) [file pone.0060889.s008.pdf]

Table S1. The TRC collection of shRNA lentiviral clones targeting human CSK

Plate: AAT74_CLO

Row	Column	Clone Name	Clone ID	Region	Target Sequence	Clone Status	Validation*
D	5	NM_004383.1-1503s1c1	TRCN0000199018	CDS	CCACTAAGTCTGACGTGTGGA	cloned	32%
D	6	NM_004383.1-592s1c1	TRCN0000199842	CDS	CCCAGCCAACTACGTCCAGAA	cloned	36%
D	7	NM_004383.1-1916s1c1	TRCN0000199031	3UTR	CCGTCTCTCTTGGACCCACCT	cloned	45%
D	8	NM_004383.1-1648s1c1	TRCN0000199500	CDS	GCCCGCAGTCTATGAAGTCAT	cloned	9%
D	9	NM_004383.1-1538s1c1	TRCN0000195031	CDS	CTCTGGGAAATCTACTCCTTT	cloned	26%

Plate: VAL02_CLO

Row	Column	Clone Name	Clone ID	Region	Target Sequence	Clone Status	Validation*
C	12	NM_004383.x-1057s1c1	TRCN0000009996	CDS	AGGGAACAAAGTCGCCGTCAA	cloned	37%
D	1	NM_004383.x-1077s1c1	TRCN0000010003	CDS	AGTGCATTAAGAACGACGCCA	cloned	84%
D	2	NM_004383.x-633s1c1	TRCN0000010007	CDS	GTACCAAACTCAGCCTCATGC	cloned	17%
D	3	NM_004383.x-876s1c1	TRCN0000010008	CDS	ACTACACCTCAGACGCAGATG	cloned	10%
D	4	NM_004383.x-903s1c1	TRCN0000010009	CDS	GTACGCGCCTCATTAAACCAA	cloned	13%
